# Haplotype analysis of the internationally distributed *BRCA1* c.3331_3334delCAAG founder mutation reveals a common ancestral origin in Iberia

**DOI:** 10.1186/s13058-020-01341-3

**Published:** 2020-10-21

**Authors:** Anna Marie De Asis Tuazon, Paul Lott, Mabel Bohórquez, Jennyfer Benavides, Carolina Ramirez, Angel Criollo, Ana Estrada-Florez, Gilbert Mateus, Alejandro Velez, Jenny Carmona, Justo Olaya, Elisha Garcia, Guadalupe Polanco-Echeverry, Jacob Stultz, Carolina Alvarez, Teresa Tapia, Patricia Ashton-Prolla, Brazilian Familial Cancer Network, Barbara Alemar, Cristina Brinckmann Oliveira Netto, Dirce Maria Carraro, Fernando Regla Vargas, Gustavo Stumpf da Silva, Ivana Lúcia Oliveira Nascimento, Kelly Rose Lobo de Souza, Maria Isabel Achatz, Miguel Angelo Martins Moreira, Maria Betânia Torrales, Maristela Pimenta, Taisa Manuela Bonfim Machado-Lopes, Ana Vega, Conxi Lazaro, Eva Tornero, Cristina Martinez-Bouzas, Mar Infante, Miguel De La Hoya, Orland Diez, Brian L. Browning, COLUMBUS Consortium, Fernando Bolaños, Raúl Murillo, Yesid Sánchez, Carolina Sanabria, Martha Lucia Serrano, John Jairo Suarez, Bruce Rannala, Manuel R. Teixeira, Pilar Carvallo, Magdalena Echeverry, Luis G. Carvajal-Carmona

**Affiliations:** 1 Genome Center, University of California Davis, Davis, CA USA; 2 Universidad del Tolima, Ibague, Colombia; 3 Hospital Pablo Tobon Uribe, Medellín, Colombia; 4 Dinamica IPS, Medellín, Colombia; 5 Hospital Universitario Hernando Moncaleano Perdomo, Neiva, Colombia; 6 Pontificia Universidad Católica de Chile, Santiago, Chile; 7 Department of Genetics, Federal University of Rio Grande do Sul (UFRGS), Porto Alegre, Brazil; 8 Post-graduate Course in Genetics and Molecular Biology, UFRGS, Porto Alegre, Brazil; 9 Medical Genetics Service, Hospital de Clinicas de Porto Alegre (HCPA), Porto Alegre, Brazil; 10 Fundación Pública Galega de Medicina Xenómica, Grupo de Medicina Xenómica-USC, CIBERER, IDIS, Santiago de Compostela, Spain; 11 Hereditary Cancer Program, Catalan Institute of Oncology, Oncobell Program-IDIBELL, Hospitalet de Llobregat, Barcelona, Spain; 12 Centro de Investigación Biomédica en Red de Cáncer (CIBERONC), Madrid, Spain; 13 Hospital Universitario Cruces, Barakaldo, Spain; 14 Cancer Genetics Group, Institute of Genetics and Molecular Biology (UVa-CSIC), Valladolid, Spain; 15 Laboratorio de Oncología Molecular, Hospital Clínico San Carlos. IdISSC (Instituto de Investigación Sanitaria San Carlos), Madrid, Spain; 16 Grupo de Cáncer Hereditario, Instituto Oncológico Vall d’Hebron (VHIO), Madrid, Spain; 17 Department of Medicine, Division of Medical Genetics, University of Washington, Seattle, WA USA; 18 Department of Evolution and Ecology, University of California Davis, Davis, CA USA; 19 Portuguese Oncology Institute of Porto (IPO Porto) and Biomedical Sciences Institute (ICBAS), University of Porto, Porto, Portugal; 20 Division de Investigaciones, Fundacion de Genética y Genómica, Ibague, Colombia; 21 University of California Davis Comprehensive Cancer Center, Sacramento, CA, USA; 22 Department of Biochemistry and Molecular Medicine, University of California Davis, Sacramento, CA, USA

**Keywords:** Breast cancer, Haplotype, *BRCA1* c.3331_3334delCAAG, Founder mutation

## Abstract

**Background:**

The *BRCA1* c.3331_3334delCAAG founder mutation has been reported in hereditary breast and ovarian cancer families from multiple Hispanic groups. We aimed to evaluate *BRCA1* c.3331_3334delCAAG haplotype diversity in cases of European, African, and Latin American ancestry.

**Methods:**

BC mutation carrier cases from Colombia (*n* = 32), Spain (*n* = 13), Portugal (*n* = 2), Chile (*n* = 10), Africa (*n* = 1), and Brazil (*n* = 2) were genotyped with the genome-wide single nucleotide polymorphism (SNP) arrays to evaluate haplotype diversity around *BRCA1* c.3331_3334delCAAG. Additional Portuguese (*n* = 13) and Brazilian (*n* = 18) BC mutation carriers were genotyped for 15 informative SNPs surrounding *BRCA1*. Data were phased using SHAPEIT2, and identical by descent regions were determined using BEAGLE and GERMLINE. DMLE+ was used to date the mutation in Colombia and Iberia.

**Results:**

The haplotype reconstruction revealed a shared 264.4-kb region among carriers from all six countries. The estimated mutation age was ~ 100 generations in Iberia and that it was introduced to South America early during the European colonization period.

**Conclusions:**

Our results suggest that this mutation originated in Iberia and later introduced to Colombia and South America at the time of Spanish colonization during the early 1500s. We also found that the Colombian mutation carriers had higher European ancestry, at the BRCA1 gene harboring chromosome 17, than controls, which further supported the European origin of the mutation. Understanding founder mutations in diverse populations has implications in implementing cost-effective, ancestry-informed screening.

## Background

Breast cancer (BC) remains the most common form of cancer and the second leading cause of cancer death among women and about 5–10% have hereditary breast cancer, explained by genetic susceptibility [1, 2]. Germline mutations in the tumor suppressor gene *BRCA1* account for the largest proportion of BC susceptibility to date and confer a 55–65% lifetime risk of developing breast cancer [2, 3]. *BRCA1* has a very heterogeneous mutation spectrum, often having high frequency of founder mutations in isolated populations such as the Ashkenazi Jewish or the Icelandic population, where few founder mutations account for most *BRCA1* carriers [4, 5].

Among Hispanic populations from Iberia and the Americas, *BRCA1* c.3331_3334delCAAG (Breast Cancer Information Core designation: 3450del4 or rs80357903) is one of the most widely distributed founder mutation and reaches its highest frequency in admixed populations from Central Colombia [6]. *BRCA1* c.3331_3334delCAAG was first described in a Canadian BC family [7], and since then reported in Europe, Latin American, the Middle Eastern, and North African patients [8–15]. The occurrence of *BRCA1* c.3331_3334delCAAG in different populations may be indicative of a mutational hotspot associated with multiple origins or a founder effect from a single ancient mutation. Although haplotype analysis has been carried out for *BRCA1* c.3331_3334delCAAG in some of these countries, they have been limited to a few intragenic markers and to a limited number of populations, often using a single individual from a carrier family [8–10]. Moreover, the *BRCA1* c.3331_3334delCAAG mutation haplotype has not been assessed on an international scale, and the ancestral origin of *BRCA1* c.3331_3334delCAAG remains to be determined. To gain insights into its origin, extensive haplotype analysis of *BRCA1* c.3331_3334delCAAG was completed in carriers from six different countries, and the age of the mutation was estimated in Colombia and Iberia. We utilized genome-wide and targeted SNP data followed by imputation, haplotype phasing, linkage disequilibrium analyses, genetic admixture estimation, and mutation dating to comprehensively assess genetic variation, spanning the entire chromosome 17, where *BRCA1* resides. Our results indicated that *BRCA1* c.3331_3334delCAAG had a single origin in Iberia.

## Materials and methods

### Study populations

#### Mutation carriers

The study was carried out using de-identified samples from of 89 *BRCA1* c.3331_3334delCAAG mutation carrier BC cases from Colombia (*n* = 32 cases from Ibague and Neiva), Spain (*n* = 13), Portugal (*n* = 16), one of which that originated in Angola (a former Portuguese colony), Chile (*n* = 10), and Brazil (*n* = 18). Mutation carriers were previously ascertained as part of population studies (Colombia, Chile and Brazil) or through high-risk hereditary cancer clinics (Spain and Portugal) [10, 11, 13–16] where all individuals signed informed consent forms and were recruited with locally approved research and clinical testing protocols.

### Genotyping and quality control procedures

#### Array genotyping

Sixty mutation carriers were genotyped with Affymetrix Axiom Human UK Biobank single nucleotide polymorphism (SNP) arrays. Samples with genotyping call rates < 95% were excluded. Basic quality control (for genotypes and missingness per individual) was completed by filtering markers with a genotype rate less than 95%, minor allele frequency ≤ 0.05, and Hardy-Weinberg equilibrium ≤ 0.00001. In total, 52 of the 60 samples passed all QC procedures.

#### Individual SNP genotyping

As additional 31 mutation Brazilian and Portuguese *BRCA1* c.3331_3334delCAAG carriers became available for our study after we completed the SNP genotyping, we decided to carry out targeted genotyping of 15 SNPs around *BRCA1* (seven and eight markers on each side of the gene, Supplementary Table 1) that were informative as they had high heterozygosity, were roughly equally spaced around the minimally shared haplotype, and had high call rates in the SNP arrays. These markers were individually genotyped with the KASP allele-specific genotyping system (LGC Genomics, London, England) following the manufacturer’s protocol and in reactions that included non-template controls, two *BRCA1* c.3331_3334delCAAG carriers (positive controls) and two *BRCA1* c.3331_3334delCAAG non-carriers (mutation negative controls). A summary of mutation carriers and genotype data are detailed in Supplementary Table 2.

#### Control SNP array data

Data available with the same SNP array on 886 Colombian control matched with cases by sex and geographical origin, were also available for analysis in this study. In addition, for genetic admixture analyses, we used publicly available genotype data from the 1000Genomes study.

### Haplotype reconstruction and IBD analysis

All analyses were carried out using GRCh37/hg19 chromosomal positions. Single nucleotide markers (SNPs) on chromosome 17, used to obtain the haplotype that flanks the *BRCA1* c.3331_3334delCAAG mutation, were phased using SHAPEIT [17] with the dataset of 938 (886 controls and 52 mutation carriers that passed genotyping QC) unrelated samples. Following phasing, BEAGLE 4.0 was used for detection of segments that were IBD [18, 19]. The ibdtrim parameter, which specifies the number of markers in a 0.15-cM region, was set to 29 for chromosome 17. The lengths of the shared haplotype segments were calculated based on a previous study by Marroni et al. [20], calculated as the sum of the distance to the last marker on either side of the *BRCA1* mutation where all mutation carriers had identical alleles. These IBD segments were verified in parallel using GERMLINE [21] as an alternative approach.

### Phylogenetic analysis of mutation haplotypes

The distance from one individual to another was determined by subtracting the distance shared from the length of chromosome 17. A phylogenetic tree was then constructed utilizing the genetic distance between mutation carriers with the UPGMA algorithm, which was incorporated in Clustal Omega [22]. This tool utilizes bootstrap analysis of 1000 replications to assess the statistical confidence in the branching order of the phylogenetic tree. SplitsTree 4.0 was used for visualization (www.splitstree.org/).

### Estimating the age of *BRCA1* c.3331_3334delCAAG in Iberia and Colombia

Sixty SNPs in a 4.34-Mb region flanking *BRCA1* (chr17: 39040105- 43387103) were selected for mutation dating. These markers captured the margins of the different mutation haplotypes determined from IBD analysis, where recombination events were observed. The DMLE+ 2.3 software [23], developed by co-author BR, was used to estimate the age of *BRCA1* c.3331_3334delCAAG. The DMLE+ 2.3 algorithm exploits an intra-allelic coalescent model to assess the linkage disequilibrium across the marker set coupled to marker locations, population growth rates, and an estimate for the proportion of the disease-bearing chromosomes. For mutation dating analyses, we focused these analyses in Colombia and Iberia as we had the highest number of available carriers and controls from these regions. For Colombia, 28 *BRCA1* c.3331_3334delCAAG carriers and 265 region-matched controls (from Neiva, where the mutation reaches its highest frequency) were used for mutation dating. From Iberia, all Spanish and Portuguese mutation carriers (*n* = 15) and 162 IBS controls (from 1000 Genomes [24]) were used for mutation dating in the peninsula. The population growth rate was estimated as previously reported in Colombia and other parts of the world [25, 26]. Map distances were estimated on the basis of physical distances given by the genetic map HapMap Phase 3.

Colombia is the country with the highest prevalence of the *BRCA1* c.3331_3334delCAAG mutation (~ 3%) in unselected breast cancer cases [6, 8, 15], and considering the breast cancer incidence, the proportion of mutation-carrying chromosomes is estimated. The proportion of mutation-carrying chromosomes sampled from Colombia was estimated to be a minimum of *f* = 0.000012 (assuming an overall prevalence of *BRCA1* carriers of 0.045) and a maximum of *f* = 0.00056 (assuming an overall prevalence of *BRCA1* carriers of 0.001). Given the prevalence of *BRCA1* carriers of about 1:1000 in the general population and using 46 million as the population of Spain, the proportion of mutation-carrying chromosomes was estimated as *f* = 0.00026 for Spain [27].

Growth rate by generation was estimated with the following equation:

$$ {\mathrm{Growth}\ \mathrm{rate}}_{\mathrm{gen}}=\frac{\ln\ \left({P}_{\mathrm{t}}/{P}_{\mathrm{o}}\right)}{g} $$

where *P*_t_ is the current population size, *P*_o_ is the initial population size, and *g* is the number of generations between the current population size and the population size at the moment of mutation origin. The current population size of Colombia is 51 million. Assuming 521 years since the Spanish arrival and 20 years per generation gives 521/20 = 26.05 generations. Assuming 1000 founders (51 × 10^6^/1000)/(26.05) = 0.42 and assuming 100 founders (51 × 10^6^/100)/(26.05) = 0.51. We performed mutation age estimates using both values. The generation growth rate of the Spanish population was assumed to be between *d* = 0.08 and 0.11. Results were determined using 100,000 burn-in iterations with 1,000,000 iterations in total for both Colombia and Spain. Additional details of all mutation dating calculations are shown in the supplementary materials.

### Genetic ancestry estimation

#### Global ancestry

Global admixture was performed using Admixture supervised algorithm [28] bootstrapped 200 times and utilized a dataset composed of 1000Genomes super populations (Africans, American, European, East Asian, South Asian) combined with an in-house Indigenous American dataset which included Maya, Aymara, Mixtec, Quechua, Tlapanec, and Nahua. To ensure that non-admixed individuals were used in the reference dataset for Admixture, Eigenstrat PCA analysis [29] was performed on the reference dataset and individuals were plotted and filtered using 3 principal components. Only individuals clustered and on ancestral axes that displayed no admixture were included in reference datasets for Admixture and RFMix [30]. In addition, Admixture was run unsupervised with *K* = 2 to *K* = 9 on the reference dataset and global ancestries were validated. Reference individuals from the 1000Genomes superpopulations displaying no admixture were utilized in Admixture and RFMix. Statistical analysis was performed with Student’s *t* test to examine distributional differences between the ancestry of carriers and non-carriers. All values are expressed as mean ± SD. *P* < 0.05 was considered as statistically significant.

#### Local ancestry

For local ancestry estimations, samples were phased using SHAPEIT and then local ancestry was calculated using RFMix PopPhased option using same reference panels as above in EM iterations, 2 EM iterations were performed, and minimum node size of 5 was used—as per recommended settings because the number of individuals in reference populations were skewed. Chromosome 17 global ancestry was calculated using Viterbi predictions of ancestry as the sum of midpoint distances between upstream and downstream markers divided by total chromosome length for ancestral predictions. For regional ancestry plots for *BRCA1* mutation carriers, counts of Amerindian, European, and African ancestry were calculated per marker and then divided by the total number of *BRCA1* mutation carriers in the set.

## Results

### Haplotype analysis and genetic distance

**Fig. 1:**
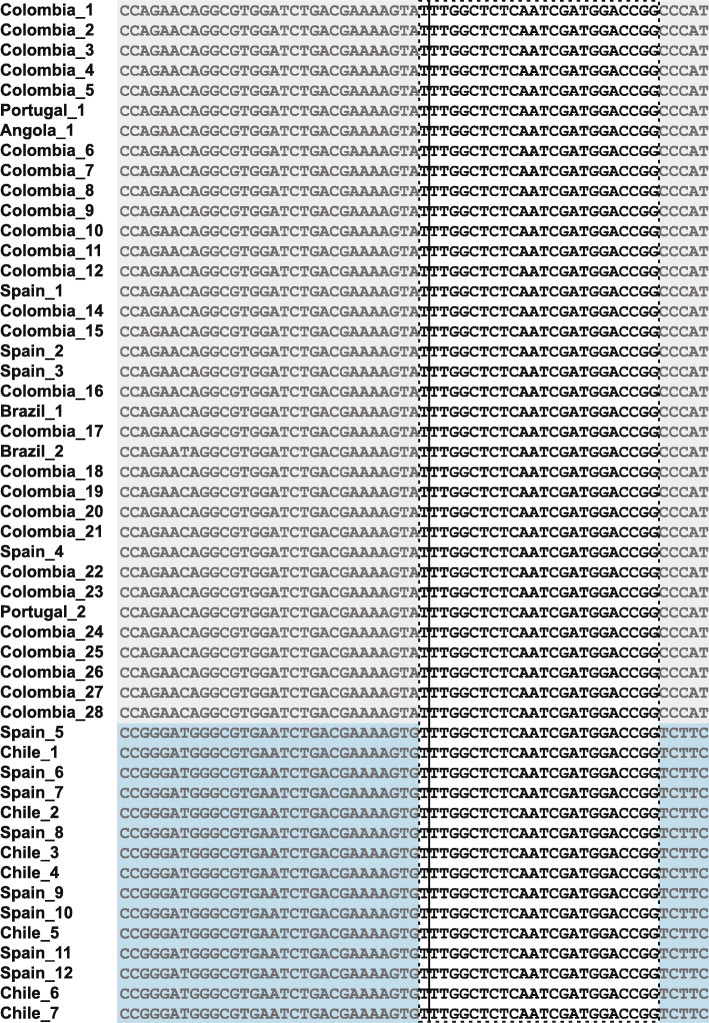
Multiple-sequence alignment of the mutation haplotype using genome-wide SNP. Data revealed a core haplotype (chr17: 41223094-41487451). The conserved region has a starting marker of Affx-13890652, and ending marker of rs75854888, creating boundaries of a 264.4-kb conserved window (dotted black box) around the mutation (location indicated by solid black line)

Using BEAGLE and GERMLINE, two main mutation haplotypes were identified among the *BRCA1* mutation carriers from the six countries (Spain, Colombia, Portugal, Angola, Brazil, and Chile). One shared haplotype was 3.9 Mb long (chr17: 39907129-43807063, between markers rs55675201 and Affx-92039463), and the other haplotype was 2.8 Mb long (chr17: 39788384-42624404, between markers rs4076033 and rs4793119). The first haplotype was shared among individuals from Colombia, Angola, Portugal, Brazil, and Spain, while the latter was shared only between Chile and Spain. Manual inspection of the mutation region via multiple-sequence alignment revealed a conserved haplotype among all mutation carriers, which was likely too small to detect using the BEAGLE or GERMLINE software. This core mutation haplotype, as determined by BEAGLE (chr17: 41223094-41487451), was flanked by Affx-13890652 and rs75854888, creating boundaries of a 264.4-kb conserved region (Fig. 1).

**Fig. 2:**
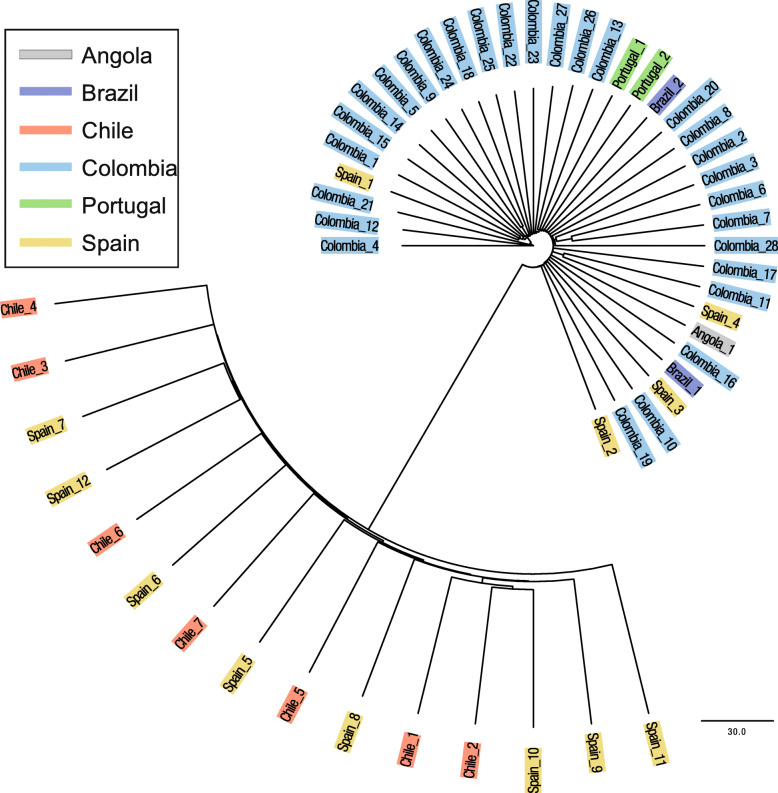
Phylogenetic tree as calculated by genetic distance of mutation haplotype between carriers. Two main mutation haplotypes exist among the mutation carriers, both of which encompass individuals from Iberia. Haplotype 1 harbors carriers from Colombia, Spain (predominantly from Catalonia), Angola, Portugal, and Brazil, while haplotype 2 harbors carriers from only Spain and Chile. An early recombination event in Spain likely occurred, as indicated by the two haplotypes sharing Spanish cases

The largest shared mutation haplotypes were identified among individuals from Colombia (26.5 Mb, chr17: 32835986-59366049, between rs75535552 and rs7215706), while the smallest were between carriers from the Iberian Peninsula. This suggests that the mutation first originated in Iberia as the length of the ancestral haplotype around the mutation is inversely correlated with the number of generations since it first appeared. The phylogenetic tree of the haplotypes was consistent with the previous analysis, where two main haplotypes exist among the mutation carriers. The mutation haplotype likely diverged in Spain prior to the mutation migrating to the other countries (Fig. 2).

### Portuguese and Brazilian population mutation haplotype

**Fig. 3:**
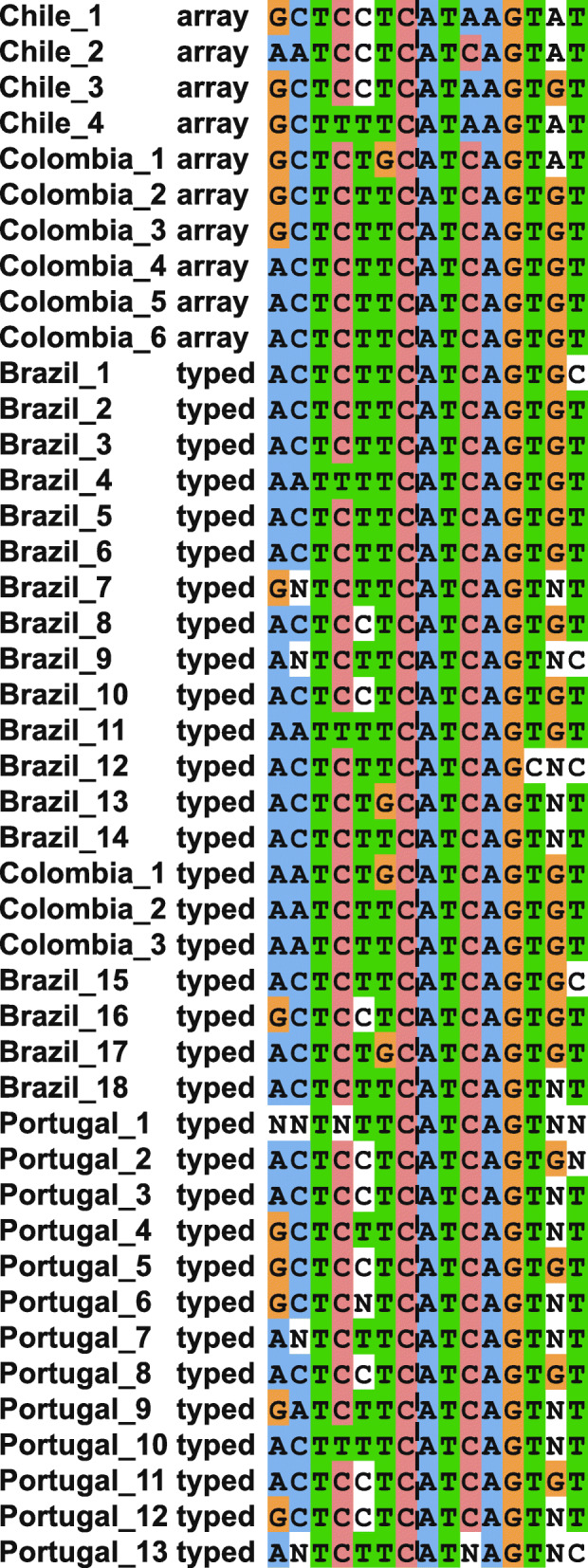
Haplotypes in 34 *BRCA1* c.3331_3334delCAAG carriers genotyped with 15 flanking SNPs. Black dashed line indicates the location of the mutation

To verify a shared haplotype among additional Portuguese and Brazilian mutation carriers which became available after SNP genotyping was completed, we genotyped these individuals with 15 SNPs surrounding the *BRCA1* c.3331_3334delCAAG mutation (Fig. 3). These mutation carriers harbored a conserved mutation haplotype that spanned from rs2229611 to rs7214920 (Chr17:41,063,466-45,051,129), indicating a minimum shared haplotype of 3.9 Mb. In the event that recombination may have occurred within this large window between markers, the two closest flanking markers rs2229611 and rs17599948 (Chr17:41,063,466-41,353,410) to the mutation produced a ~ 290-kb shared window.

### Estimating chromosome 17 European ancestry among Colombian mutation carriers

**Fig. 4:**
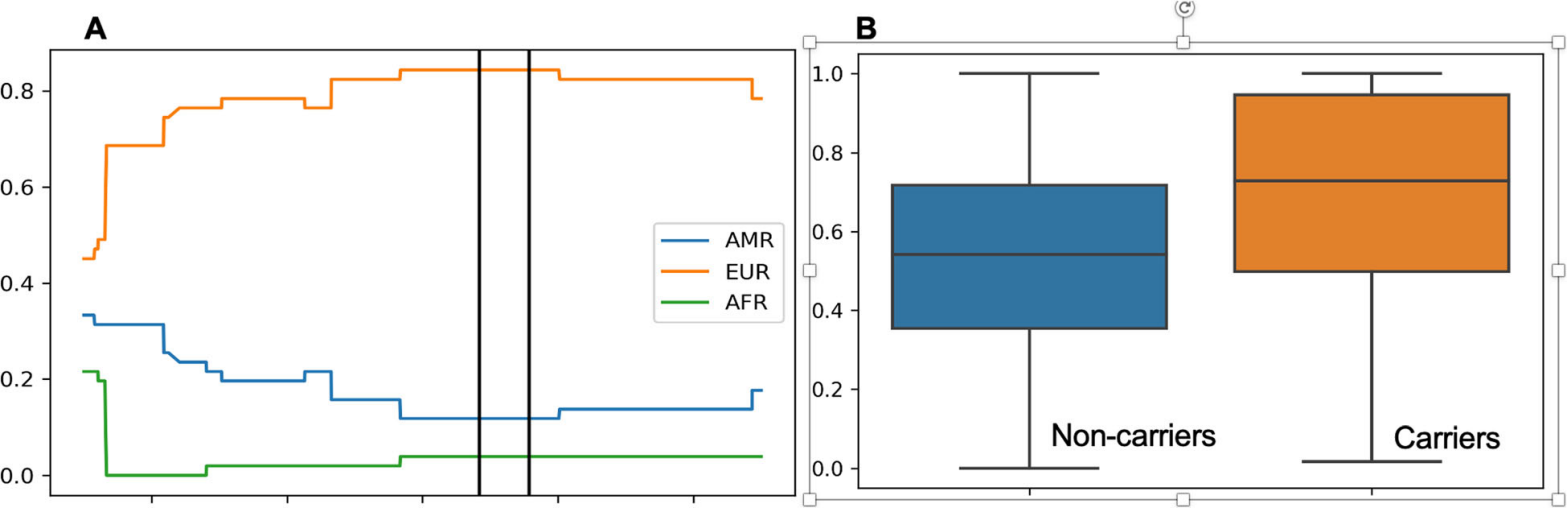
**a** Fractions of local American, European, and African ancestry (*y* axis) on chromosome 17 (*x* axis), with two vertical bars indicating the *BRCA1* region from Fig. 1. **b** Chromosome European ancestry (*y* axis) among Colombian non-mutation carriers (left) and mutation carriers (right)

Given that the mutation likely originated from Spain, we hypothesized that Colombian carriers would be on average, more European along chromosome 17, where BRCA1 locates, than the average Colombian controls. We found that local ancestry among carriers was higher in the *BRCA1* region (Fig. 4a) and that mutation carriers had higher chromosome 17 European ancestry than non-mutation carriers (*P* = 0.000116, Fig. 4b).

### Estimation of allele age in Iberia and Colombia

To estimate the date of the mutation, 60 SNPs residing within a 4.35-Mb window around the *BRCA1* c.3331_3334delCAAG mutation were chosen to be used with the DMLE approach. For Colombia, the mutation age estimates in generations (posterior mean and 95% credibility interval) with *f* = 0.000012 were 36.3 (31.3, 44.3) assuming *d* = 0.42 and 29.7 (25.4, 36.8) assuming *d* = 0.51. With *f* = 0.00056, the estimates were 27.6 (22.5, 36.3) assuming *d* = 0.42 and 24.8 (19.9, 32.3) assuming *d* = 0.51. Assuming 20 years per generation, these mean ages range from 496 to 726 years. For Iberia, using *f* = 0.00026, the mutation age estimates were 121.0 (97.1, 153.6) assuming *d* = 0.08 and 98.0 (75.9, 128.9) assuming *d* = 0.11. Assuming 20 years per generation, these mean ages range from 1960 to 2400 years. These results support the hypothesis that one or a small number of copies of the *BRCA1* mutation were introduced into Colombia via Spanish colonists at the time of the population founding/admixture event.

## Discussion

The comparison of haplotypes between individuals with the same mutation can distinguish whether high-frequency alleles derive from an older or more recent single mutational event and can also determine whether the mutation had arisen independently from multiple individuals. Our study suggests that the *BRCA1* c.3331_3334delCAAG was introduced to Colombia and South America early in the colonization of the country, resulting in a high mutation prevalence in the population. The estimated age of this mutation in Colombia is consistent with this historical account.

Haplotype length is inversely correlated with the number of generations separating the common ancestor from cases with the mutation in the present time. Our approach revealed a shared mutation haplotype by carriers of six countries, multiple continents, and numerous families. These findings depict a history of immigration that is consistent with ancestral links between these populations. The estimated ages from our study and ancestry estimates in Colombian mutation carriers are consistent with the country’s history and origin of the mutation, in addition to the genetic demography of Colombia. The mutation was likely introduced to the region during early colonial times during the early 1500s, and our findings in Iberia are consistent with previous dating estimates for other mutations [31]. Moreover, our studies suggest an early recombination event in Spain, which results in the two main haplotypes around the mutation. Spanish and Portuguese colonization of Brazil, Chile, and Colombia during the early 1500s is consistent with the mutation distribution found in our study. In fact, the differences in time periods of Spanish colonization and conquest can be represented by the two main mutation haplotypes found in this study. Interestingly, we also found the same haplotype in a carrier from Angola, a former Portuguese colony, and thus our findings are consistent with the European colonization of Africa and the Americas.

We used genome-wide SNP data to capture the mutation haplotype and estimate mutation age rather than traditional microsatellite markers, which allowed us to comprehensively assess the mutation haplotype via IBD analysis and multiple sequence alignment. A similar approach can be exploited for mapping new variants [32]. We recognize that there may be more to explore surrounding this mutation. While we were able to date the mutation in Iberia and Colombia, we lacked sufficient control data for other countries, such as Chile or Brazil, to allow us to date the mutation in such countries. We anticipate that the mutation age in the other countries will be related to the time of Spanish and Portuguese colonization. We also cannot exclude that the mutation may have multiple ancestral origins in countries without a history of colonization by those countries, such as Canada or Norway, where this mutation has been also reported [7, 33]. Furthermore, while our study in Colombia focused on communities from the central Andean region, where we have shown that they have a predominant European and Indigenous American ancestry [16, 34–40], a recent study in Afro-Colombian populations from the west of the country also identified *BRCA1* c.3331_3334delCAAG carriers, which may suggest additional origins in other Colombian groups [41]. A similar analysis with carriers from these populations would be necessary to confirm this hypothesis.

## Conclusions

In summary, we demonstrated the existence of a single ancestral mutation haplotype among six different countries and general mutation age in the Colombian and Iberian populations are in agreement with historic migration and cultural patterns. Colombian mutation carriers have a higher European ancestry than non-mutation carrier cases, a finding that further support a European origin of *BRCA1* c.3331_3334delCAAG. We also highlight the advantage of utilizing genomic approaches to comprehensively assess founder mutations, since genome-wide SNP data can be exploited to measure ancestry or genetic distance between mutation haplotypes, in addition to haplotype analysis and mutation age estimation.

## Supplementary information

**Additional file 1: Supplementary Table 1.** KASP primers used to type mutation haplotype in *BRCA1* c.3331_3334delCAAG mutation carriers from Portugal and Brazil. **Supplementary Table 2.** Summary of mutation carriers and genotype experiments.

**Additional file 2.**

## Data Availability

All data generated or analyzed during this study are included in this published article [and its supplementary information files].

## Abbreviations

BC: Breast cancer
SNP: Single nucleotide polymorphism
IBD: Identical by descent
IBS: Iberian populations in Spain
DMLE+: Bayesian linkage disequilibrium gene mapping
