# Supplementary material for: Haplotype analysis of the internationally distributed BRCA1 c.3331_3334delCAAG founder mutation reveals a common ancestral origin in Iberia
Source: Breast Cancer Res. 2020 Oct 21;22:108. doi: 10.1186/s13058-020-01341-3 (PMC7579869; doi:10.1186/s13058-020-01341-3)
Supplement: Supplementary file 1 — Additional file 1: Supplementary Table 1. KASP primers used to type mutation haplotype in BRCA1 c.3331_3334delCAAG mutation carriers from Portugal and Brazil. Supplementary Table 2. Summary of mutation carriers and genotype experiments. [file 13058_2020_1341_MOESM1_ESM.docx]

**Supplementary Table 1:** KASP primers used to type mutation haplotype in *BRCA1* c.3331_3334delCAAG mutation carriers from Portugal and Brazil.

| **SNP** | **A1** | **A2** | **Cohort MAF** | **3450del4 (-) MAF** | **3450del (+) MAF** | **Difference in MAF** | **Chr17 coordinate** | **Distance from Mutation (Mb)** |  |
| --- | --- | --- | --- | --- | --- | --- | --- | --- | --- |
| **rs936656** | A | G | 0.3399 | 0.3373 | 0.4535 | 0.1162 | 33433819 | -7.810144 |  |
|  |  |  | **Primer_A1** | GAAGGTGACCAAGTTCATGCTGGGAGGGTTTCCTGGAAGAATCA | | | | | |
|  |  |  | **Primer_A2** | GAAGGTCGGAGTCAACGGATTGGAGGGTTTCCTGGAAGAATCG | | | | | |
|  |  |  | **Primer_C1** | CCCTGTTGATGCCATCATCCCATTT | | | | | |
| **rs7211602** | A | C | 0.2764 | 0.2728 | 0.4318 | 0.159 | 36026400 | -5.217563 |  |
|  |  |  | **Primer_A1** | GAAGGTGACCAAGTTCATGCTGGCAACATTTTCAAGAGGGACATTG | | | | |  |
|  |  |  | **Primer_A2** | GAAGGTCGGAGTCAACGGATTAGGCAACATTTTCAAGAGGGACATTT | | | | |  |
|  |  |  | **Primer_C1** | CCAGACTTCTCACTATCCTGGTCAT | | | | |  |
| **rs16966483** | C | T | 0.06497 | 0.06179 | 0.2045 | 0.14271 | 36740607 | -4.503356 |  |
|  |  |  | **Primer_A1** | GAAGGTGACCAAGTTCATGCTCCCTGTGGCTTGAATCTCGGA | | | | |  |
|  |  |  | **Primer_A2** | GAAGGTCGGAGTCAACGGATTCCCTGTGGCTTGAATCTCGGG | | | | |  |
|  |  |  | **Primer_C1** | TTCTCCAAGGAACCTCAGTGTGCAA | | | | |  |
| **rs7217037** | T | C | 0.3657 | 0.3665 | 0.3295 | -0.037 | 38223190 | -3.020773 |  |
|  |  |  | **Primer_A1** | GAAGGTGACCAAGTTCATGCTCACATATACAGTTTGTTATGAACTAG | | | | |  |
|  |  |  | **Primer_A2** | GAAGGTCGGAGTCAACGGATTGCTCACATATACAGTTTGTTATGAACTAA | | | | |  |
|  |  |  | **Primer_C1** | CCTCATCTGTAAATTGGGGATGATCATAT | | | | |  |
| **rs17558560** | T | C | 0.3096 | 0.3037 | 0.4318 | 0.1281 | 38936658 | -2.307305 |  |
|  |  |  | **Primer_A1** | GAAGGTGACCAAGTTCATGCTCATCCAGGACTCTTCGCAAACC | | | | |  |
|  |  |  | **Primer_A2** | GAAGGTCGGAGTCAACGGATTCATCCAGGACTCTTCGCAAACT | | | | |  |
|  |  |  | **Primer_C1** | CTAAGGTTTGAAAACGAGCTAGCGCTT | | | | |  |
| **rs11651246** | G | T | 0.1851 | 0.1802 | 0.3977 | 0.2175 | 40759936 | -0.484027 |  |
|  |  |  | **Primer_A1** | GAAGGTGACCAAGTTCATGCTAAGAGACCCAGTTAAAGGTGTCACA | | | | |  |
|  |  |  | **Primer_A2** | GAAGGTCGGAGTCAACGGATTGAGACCCAGTTAAAGGTGTCACC | | | | |  |
|  |  |  | **Primer_C1** | GCTAAAGGCTCTGGAAGCCACAAAT | | | | |  |
| **rs2229611** | T | C | 0.4103 | 0.415 | 0.2045 | -0.2105 | 41063465 | -0.180498 |  |
|  |  |  | **Primer_A1** | GAAGGTGACCAAGTTCATGCTAAGAGATGTGGAGTCTTCGGTGTTT | | | | |  |
|  |  |  | **Primer_A2** | GAAGGTCGGAGTCAACGGATTGAGATGTGGAGTCTTCGGTGTTC | | | | |  |
|  |  |  | **Primer_C1** | GTAGTCCTCCTCAATCCCTGGCAT | | | | |  |
| **rs80357903** | CAAG | - |  |  |  |  | 41244214 | 0 |  |
| **rs17599948** | G | A | 0.2452 | 0.2484 | 0.1023 | -0.1461 | 41353409 | 0.109446 |  |
|  |  |  | **Primer_A1** | GAAGGTGACCAAGTTCATGCTGGAAAATGCTGGAGCCTTGACTA | | | | |  |
|  |  |  | **Primer_A2** | GAAGGTCGGAGTCAACGGATTGGAAAATGCTGGAGCCTTGACTG | | | | |  |
|  |  |  | **Primer_C1** | CGGAAATGTCTAGCTTTTGGGGAGAA | | | | |  |
| **rs1107748** | C | T | 0.4158 | 0.4199 | 0.2386 | -0.1813 | 41773813 | 0.52985 |  |
|  |  |  | **Primer_A1** | GAAGGTGACCAAGTTCATGCTAAATGACAATAGTTCTTTCCTAGACTCAT | | | | |  |
|  |  |  | **Primer_A2** | GAAGGTCGGAGTCAACGGATTATGACAATAGTTCTTTCCTAGACTCAC | | | | |  |
|  |  |  | **Primer_C1** | TCTTTACAAGGGCACTGCAGGCAAA | | | | |  |
| **rs8077889** | C | A | 0.1526 | 0.1456 | 0.4545 | 0.3089 | 41878165 | 0.634202 |  |
|  |  |  | **Primer_A1** | GAAGGTGACCAAGTTCATGCTAATCATTTTTGCTCAGAGACAACCCA | | | | |  |
|  |  |  | **Primer_A2** | GAAGGTCGGAGTCAACGGATTATCATTTTTGCTCAGAGACAACCCC | | | | |  |
|  |  |  | **Primer_C1** | CGTCACTGTAAACCATTTCTGTCAAGAAT | | | | |  |
| **rs3826425** | G | A | 0.2363 | 0.2387 | 0.1279 | -0.1108 | 42981653 | 1.73769 |  |
|  |  |  | **Primer_A1** | GAAGGTGACCAAGTTCATGCTATGGTGGCCACTTTCCAGGACA | | | | |  |
|  |  |  | **Primer_A2** | GAAGGTCGGAGTCAACGGATTGGTGGCCACTTTCCAGGACG | | | | |  |
|  |  |  | **Primer_C1** | GAAGACATGGAGGTGCCCATAGTAT | | | | |  |
| **rs7214920** | A | G | 0.1215 | 0.118 | 0.2791 | 0.1611 | 45051128 | 3.807165 |  |
|  |  |  | **Primer_A1** | GAAGGTGACCAAGTTCATGCTTGACAAGAATCTGCACTCAAGAGATA | | | | |  |
|  |  |  | **Primer_A2** | GAAGGTCGGAGTCAACGGATTGACAAGAATCTGCACTCAAGAGATG | | | | |  |
|  |  |  | **Primer_C1** | GTCGCACGCATGCACACACATTTAA | | | | |  |
| **rs16957364** | C | T | 0.05983 | 0.05651 | 0.2045 | 0.14799 | 46555744 | 5.311781 |  |
|  |  |  | **Primer_A1** | GAAGGTGACCAAGTTCATGCTATTTGCACACGGTGGCCACAA | | | | |  |
|  |  |  | **Primer_A2** | GAAGGTCGGAGTCAACGGATTATTTGCACACGGTGGCCACAG | | | | |  |
|  |  |  | **Primer_C1** | TGCCCAGCTGCACTGGTTTCTTAA | | | | |  |
| **rs16948048** | G | A | 0.2686 | 0.2641 | 0.4659 | 0.2018 | 47440465 | 6.196502 |  |
|  |  |  | **Primer_A1** | GAAGGTGACCAAGTTCATGCTCCCTGTACCCTAAACACTTTCAAC | | | | |  |
|  |  |  | **Primer_A2** | GAAGGTCGGAGTCAACGGATTCTCCCTGTACCCTAAACACTTTCAAT | | | | |  |
|  |  |  | **Primer_C1** | GTAGCATTTATCCGAGTTCAGAGACAA | | | | |  |
| **rs9303562** | C | T | 0.3278 | 0.3273 | 0.3523 | 0.025 | 48857042 | 7.613079 |  |
|  |  |  | **Primer_A1** | GAAGGTGACCAAGTTCATGCTAAATAGAAACACCTCTCAGCCCG | | | | |  |
|  |  |  | **Primer_A2** | GAAGGTCGGAGTCAACGGATTACAAATAGAAACACCTCTCAGCCCA | | | | |  |
|  |  |  | **Primer_C1** | CCATTGATTATGACATGAACTTCTGAGGAA | | | | |  |

**Supplementary Table 2:** Summary of mutation carriers and genotype experiments

| **Country** | **Cases with**  **mutation (*n*)** | **Cases with Affymetrix Array Data (*n*)** | **Cases with KASP Genotype Data (*n*)** |
| --- | --- | --- | --- |
| Angola | 1 | 1 | 0 |
| Brazil | 18 | 2 | 18 |
| Colombia | 32 | 32 | 3 |
| Chile | 10 | 10 | 0 |
| Portugal | 15 | 2 | 13 |
| Spain | 13 | 13 | 0 |
