# Supplementary material for: Haplotype analysis of the internationally distributed BRCA1 c.3331_3334delCAAG founder mutation reveals a common ancestral origin in Iberia
Source: Breast Cancer Res. 2020 Oct 21;22:108. doi: 10.1186/s13058-020-01341-3 (PMC7579869; doi:10.1186/s13058-020-01341-3)
Supplement: Supplementary file 2 — Additional file 2. [file 13058_2020_1341_MOESM2_ESM.pdf]

The sample fraction of disease chromosomes (f)

Slatkin and Rannala [1] assumed a model with a random sample of chromosomes from a population, a certain fraction of which carry the disease mutation. The sample fraction f is defined by [1] as

$$f = \frac{n}{2N} \tag{1}$$

where n is the number of chromosomes sampled and N is the diploid population size. The fraction f in the model is assumed to be a random sample. However, disease chromosomes are often ascertained (we do not randomly sample individuals but often sample individuals who are more likely to carry the disease mutation). Thus, for an ascertained sample of disease chromosomes Slatkin and Rannala [1] calculated the expected sample size n that would be needed to obtain a sample of c disease chromosomes on average. If the frequency of the disease mutation in the population is q then the expectation (average) is

$$q = \frac{c}{n},$$

and solving for n gives

$$n = \frac{c}{q}. \tag{2}$$

Substituting equation 2 into equation 1 gives the formula for f,

$$f = \frac{c}{2Nq}. \tag{3}$$

For rare mutations (population frequency less than 10%), the population mutation frequency is proportional to twice the population frequency of individuals heterozygous for the mutation.

Estimates of f for Columbia

We consider a range of plausible values for the frequency of mutation-bearing heterozygotes in the Columbian population. Assuming the frequency of heterozygotes in Columbia is 0.045 (probably an overestimate) gives $q_1 = 0.0225$ and assuming that the frequency of heterozygotes in Columbia is the same as Spain, 0.001, (probably an underestimate), gives $q_2 = 0.0005$. The current population size of Columbia is approximately $50,000,000$, thus $2N = 100,000,000$. In total $c = 26$ disease chromosomes were sampled. Substituting these values into equation 3 and rounding to a single digit gives

$$f_1 = \frac{26}{q_1 \times 100,000,000} = \frac{26}{0.0225 \times 100,000,000} = 0.000016.$$

$$f_2 = \frac{26}{q_2 \times 100,000,000} = \frac{26}{0.0005 \times 100,000,000} = 0.00052.$$

Estimates of f for Spain

Assuming that the frequency of heterozygotes in Spain is 0.001 gives $q = 0.0005$. The current population size of Spain is approximately $46,000,000$, thus $2N = 92,000,000$. In total, $c = 12$ disease chromosomes were sampled. Substituting these values into equation 3 gives

$$f = \frac{12}{0.0005 \times 92,000,000} = 0.00026.$$

Population growth rates

To calculate population growth rates we assume exponential growth for both Columbia and Spain. Assuming deterministic population growth,

$$N_t = N_0 \mathrm{e}^{rt},$$

and solving for r gives,

$$r = \frac{1}{t} \log \left(\frac{N_t}{N_0} \right).$$

Population growth rate of Columbia

The Spanish arrived in 1499 in the region that is now Columbia, 521 years ago. Assuming 20 years per generation gives $521/20 = 26.05$ generations. We calculated the population growth rate using either $N_0 = 1000$ or $N_0 = 100$ as the effective number of founders is uncertain. Using $N_0 = 1000$ gives

$$r_1 = \frac{1}{26.05} \times \log \left(\frac{50,000,000}{1000} \right) = 0.42,$$

and using $N_0 = 100$ gives

$$r_1 = \frac{1}{26.05} \times \log \left(\frac{50,000,000}{100} \right) = 0.50,$$

Population growth rate of Spain

The population of Spain increased from about 7.4 million in 1700 to about 46,000,000 in 2020. Assuming 20 years per generation this represents 16 generations and corresponds to a growth rate of

$$r = \frac{1}{16} \times \log \left(\frac{46,000,000}{7,400,000} \right) = 0.11.$$

However, Spain experienced relatively more immigration during this period than Columbia so the intrinsic rate of growth is probably considerably lower – we therefore used a minimum growth rate of 0.08 and a maximum of 0.11

References

[1] Slatkin, Montgomery, and Bruce Rannala. 1997. Estimating the age of alleles by use of intraallelic variability. American journal of human genetics 60: 447.
